# “It Is a Whole Different Life from the Life I Used to Live”: Assessing Parents’ Support Needs in Paediatric Palliative Care

**DOI:** 10.3390/children9030322

**Published:** 2022-03-01

**Authors:** Samar M. Aoun, Roswitha Stegmann, Renee Deleuil, Suzanne Momber, Lisa Cuddeford, Marianne B. Phillips, Maureen E. Lyon, Fenella J. Gill

**Affiliations:** 1 University of Western Australia, Crawley, WA 6009, Australia; 2 Perron Institute for Neurological and Translational Science, Nedlands, WA 6009, Australia; 3 Public Health Palliative Care Unit, School of Psychology and Public Health, La Trobe University, Melbourne, VIC 3086, Australia; r.stegmann@latrobe.edu.au; 4 Child and Adolescent Health Service, Perth Children’s Hospital, Nedlands, WA 6009, Australia; renee.deleuil@health.wa.gov.au (R.D.); suzanne.momber@health.wa.gov.au (S.M.); lisa.cuddeford@health.wa.gov.au (L.C.); marianne.phillips@health.wa.gov.au (M.B.P.); f.gill@curtin.edu.au (F.J.G.); 5 Children’s National Hospital, Washington, DC 20010, USA; mlyon@childrensnational.org; 6 George Washington University School of Medicine and Health Sciences, Washington, DC 20052, USA; 7 School of Nursing, Curtin University, Perth, WA 6102, Australia

**Keywords:** palliative care, end-of-life care, equity, public health approach, compassionate communities, caregiving, parents, psychosocial support

## Abstract

Aims: This feasibility study aimed to systematically identify and address the support needs of parents of children with life-limiting illnesses and to assess whether the systematic approach was acceptable and relevant to parents. Methods: The CSNAT (Paediatric) intervention consisted of two assessment visits with the paediatric palliative care team, 2–8 weeks apart, comprising conversations about sources for support in a tertiary children hospital in Western Australia (2018–2019). Audio-recorded telephone interviews were conducted with parents, and inductive thematic analysis was undertaken. Results: All 28 parents who were involved in the intervention agreed to be interviewed. Five themes summarised their experience: caregiving challenges, perceived gaps and feelings of isolation; the usefulness and practicality of the systematic assessment; emotional responses to self-reflection; feelings of validation and empowerment; and received supports responsive to their needs. Conclusions: Parents appreciated the value of this systematic approach in engaging them in conversations about their needs and solutions to address them. While clinical service support was affirmed by parents, they were left wanting in other areas of practical, psychosocial, and emotional support. Palliative care services need to build stronger partnerships with supportive community networks through compassionate communities volunteer models of care to address the non-clinical needs of these families.

## 1. Background

Paediatric palliative care (PPC) begins with the diagnosis of a child’s life-limiting illness and focuses on improving the quality of life for the child and their family [1,2,3,4]. PPC addresses physical, psychological, social, and spiritual needs for the child and their family. Families with seriously ill children face multiple and complex challenges that are individual to that family’s circumstances. Receiving individualised supportive care from health professionals and the building of effective and trusting relationships have been reported to be essential components of PPC [5], yet there may be a gap between the identified ideal approach and the actual experiences of children and their families [6]. 

Families’ support needs are not always adequately addressed by health professionals. Reported unmet needs include a lack of access to psychological support for parents and siblings of the child with a life-limiting illness [6]. Other unmet needs include a lack of access to home support and to educational supports and resources [7]. Despite the known unmet needs, there have been very few reports of a systematic approach to assess and evaluate parent caregiver support needs [8,9] and no reports of using evidence-based tools to routinely help identify needs or review whether support needs were met [7]. 

The Carer Support Needs Assessment Tool (CSNAT) is a validated tool designed to identify and address the individual support needs of caregivers of adults with life-limiting conditions [10]. The CSNAT approach involves the creation of an action plan in response to the needs assessment, with ongoing further reviews and follow-ups. The 14-item CSNAT covers two domains that address enabling the caregiver to care as well as direct support for the caregiver. The adapted 16-item paediatric version, CSNAT (Paediatric) [11], was modified from the adult version for use with parent caregivers in the PPC setting. 

While the benefits of implementing the assessment of caregiver needs into routine practice has been demonstrated in adult settings, this had not been shown in the paediatric setting. We undertook a pilot study to trial the use of the CSNAT (Paediatric) in an Australian PPC setting [12]. In designing the pilot study, we recognised that, in addition to testing initial CSNAT (Paediatric) implementation outcomes, it was also important to examine the feasibility to understand whether the CSNAT (Paediatric) was appropriate for further testing and whether it can be recommended for implementation into routine care [13]. 

Feasibility studies can address a number of focus areas and commonly include acceptability from the perspectives of individual recipients and from those involved in implementing the intervention [14,15]. 

## 2. Objectives

The objective of this qualitative study was to assess the acceptability of using the CSNAT (Paediatric), a systematic approach to caregiver needs assessment, from the perspective of parents in a paediatric palliative care setting in Western Australia.

## 3. Methods

A steering group guided the development and implementation of the project and included researchers, health professionals (HPs—palliative care consultants and specialist nurses), and two parents with lived experience of receiving paediatric palliative care services. A two-hour training session for HPs was conducted by the researchers and regular 8-weekly team meetings took place for the duration of the project to identify and discuss implementation and data collection issues. The project was approved by the Human Research Ethics Committee of the Child and Adolescent Health Service (RGS0000000772) and La Trobe University Research Ethics Committee. Participants provided written informed consent prior to the start of data collection. The standards for reporting qualitative research were followed [16].

### 3.1. Setting 

Paediatric palliative care services in Western Australia are co-ordinated from the specialist children’s hospital and provide care for children with life-limiting diseases and their families.

### 3.2. Participants 

Parents of children aged 18 years and younger receiving palliative care who could speak, read, and understand English were invited to participate (2018–2019). Parents of children who were assessed to be within 6–8 weeks of dying were excluded.

Parents participated in semi-structured telephone interviews to obtain feedback regarding their experience using the CSNAT (Paediatric). The brief interview guide was developed and pre-tested with two parents to ensure clarity of the following questions: How easy or difficult was it for you completing the CSNAT (Paediatric)? Can you please describe in what ways completing this assessment approach was helpful in getting the support you needed? In what ways did the experience of identifying your needs affect what you did yourself? In what ways did you feel that your needs as a caregiver were acknowledged and listened to in a way that was distinct from the needs of [child’s name] for whom you provide care? What improvements if any could be made to this assessment approach?

### 3.3. Description of the Intervention 

The adult version of CSNAT is a validated, evidence-based tool used to systematically identify family caregiver support needs during their relative’s end-of-life care [17]. The tool is a caregiver-led, supportive intervention facilitated by the HP. The CSNAT uses a screening format and is structured around 14 broad domains. The domains fall into two distinct groups: those enabling the caregiver to care and those that enable more direct support for the caregiver. It is brief but comprehensive and enables caregivers to identify the domains in which they require further support, which can then be discussed with health professionals through an action plan that needs to be regularly reviewed. The CSNAT (Paediatric) has two additional domains, and its detailed description is reported in [12].

### 3.4. Recruitment and Data Collection

Eligible parents with a child known to paediatric palliative care services and attending a clinical appointment were identified by the HP, provided with information about the study, and invited to participate. 

The CSNAT (Paediatric) was completed during a scheduled clinical appointment or over the telephone when deemed by the HP to be appropriate. Parents were given the option to complete the CSNAT (Paediatric) on their own or in the presence, and/or with the assistance, of the HP. Parents completed it for a second time 2–8 weeks later, either at a clinical appointment or over the telephone with the HP. Interviews took place approximately 2 weeks after the second assessment had been completed.

### 3.5. Data Analysis 

Interviews were audio-recorded and transcribed verbatim. Transcripts were imported into NVivo version 12 software for data management (QSR International Pty Ltd., Melbourne, Australia). Inductive thematic analysis was undertaken using the six phases described by Braun and Clarke [18]. Initial coding was carried out independently by two co-authors, one being the interviewer. Transcriptions were read and re-read to identify key words and phrases that were then grouped into categories labelled with codes. To enhance the credibility of the findings, the interviewer participated in the analysis process so that consideration of the nonverbal context was assured. To further ensure the trustworthiness of our findings, transferability is established by our description of the study’s setting and participants. 

## 4. Results 

### 4.1. Participant Characteristics 

In total, 33 parents agreed to be enrolled in the project, and 28 of them completed the intervention: there were 8 parents of children with cancer (code C in quotes) and 20 with non-cancer diagnoses (code NC in quotes). Most parent caregivers were female (93%) and aged from 27 to 55 years old, with a mean of 41.7 years (SD = 8.4). In addition, 82% of parents were married/de facto married and 14% were separated/divorced, and 75% of parents had an Australian background, of which one was of Aboriginal descent. Approximately 80% of parents lived with their child in the metropolitan area. Children’s ages ranged from under one to 18 years, with a median of 10 years. The median time from diagnosis was 64 months, the median time since the child first became unwell was 94 months, and the median time interval following referral to palliative care was 23 months. Other characteristics are described in more detail in [12].

Reasons for declining to participate or not completing the study were due to the child rapidly deteriorating or because the parent was feeling overwhelmed.

### 4.2. Parents’ Feedback

The interviews explored parents’ experiences using the CSNAT (Paediatric), its usefulness to them, and the support they received. All participating 28 parents agreed to be interviewed by the research officer over the telephone. The median length of interview was 16 min (range 6–45) with a mean duration of 17.6 min (SD = 7.92). 

Five themes were identified: (1) caregiving challenges, gaps, and feelings of isolation elicited by the assessment process, (2) the practicality and usefulness of systematic assessment, (3) the self-reflection evoking emotional responses, (4) the validation and empowerment experienced by being asked about their needs, (5) receiving support responsive to their needs. Table 1 summarises the five themes and their sub-themes.

**Theme 1. *Caregiving challenges and gaps in support elicited by the assessment process*.**

- *Subtheme 1.1: Mental impact on parents and perceived gaps in support*

Feedback on the assessment process triggered parents to describe their long, challenging journey through the course of the disease and the impact it had on them mentally and the perceived lack of support for their own needs.

*I actually ended up, I had a major breakdown, about 18 months into this. My marriage had already broken down and there were issues at work, a stressful job. So, with this, with [child name] I think once we went through the process of him having the surgery, chemo, radiation, more chemo, when it was sort of getting to the end of that and I realised, “oh gosh” and I recognised the fact that I was falling into a deep hole despite having, you know, medication and gone to a psychologist.*

(P07-C)

*So, there is none of that is, none of that care services are free [for parents]. You have to go and do it externally. But sometimes you even wonder if it actually would be helpful if it was done as part of the overall care for the child, because it’s really if the parents are not coping the child is not going to get what they need either.*

(P27-NC)

- *Subtheme 1.2: Feelings that parents come last*

As parents, they were putting themselves last *“Because I felt it wouldn’t be fair for the focus be on me when my child is so ill”* (P10-C), although for them major life changes happened: *“It’s changed my life, it is a whole different life from the life I used to live”* (P19-NC); *“That side of the whole caring role, the parent’s role, their life stops”* (P13-NC). Therefore, some assessment questions did not seem relevant to them when internal or external support was not forthcoming:

*When it comes to the questions where you are getting time for yourself and looking after your own needs etcetera. Obviously, we don’t really get to do that because we are parents at the same time.*

(P4-NC)

*Like at the moment I have absolutely no time for doing things for myself or socialising or anything. So, you know, there is just nothing. I tried to go out the other night and [child name] had a really bad night and I had to come home. And it was the first time in six months, I think, I am gone out. So, this was just, you know, it was a bit of a disaster. Yeah. It’s a bit disappointing.*

(P14-NC)

- *Subtheme 1.3: Frustration with inadequacies of external providers*

A number of parents of children with complex needs commented that the assessment process would not be useful because of entrenched systematic inadequacies:

*So, I would say, no it hasn’t helped. But that has nothing to do with the survey. Because of outside providers I am struggling with. Because my thing is the whole transition… I am still fighting the same fights with the same people. And getting the same frustration.*

(P25-NC)

*Sometimes a lack of understanding of the day-to-day needs that we have and it’s almost like it’s so much red tape you have to get through and it is almost like there is no common sense at the other end. You know, our child is in a wheelchair, we are having to lift him in, you know, we’ve hurt our backs doing it. So, frustration, you know, when it appears black and white to us.*

(P28-NC)

- *Subtheme 1.4: Care lacking a psychosocial focus and feelings of isolation*

The perceived lack of guidance, information, and feelings of isolation with no one to turn to for other types of support compounded negative feelings, *“The focus is much more there on the clinical side, rather than the psychological and social side”* (P11-C):

*Yeah, big gaps in, I am going to say in care. Because the child gets medical support which is amazing. There is absolutely no fault there. but there is no guidance for parents, like, you know, especially in the early days of a diagnosis.*

(P27-NC)

*Yeah, parent struggle because there is no one in your current circle to talk to. So, when you often become friends with other families in a similar situation which is great. But those families can’t really deal with your problems either because they’ve got their own. You don’t want to burden other family with your worries because they are feeling the same.*

(P30-NC)

*You do need lots of hands on, lots of people to come in, you know, such as the OT, the social worker, the doctor, the nurse, whether it’s physio required as well. I think we just sort of find out various other things just by chance, sometimes by stumbling across something, or another parent, perhaps in the waiting room talking about a particular thing.*

(P10-C)

**Theme 2. *Practicality and usefulness of the systematic assessment*.**

- *Subtheme 2.1: Straightforward form and approach, structured and comprehensive*

Parents described that the CSNAT (Paediatric) and its structured format comprehensively acknowledged their support needs. They experienced the assessment as straightforward and relevant. The systematic assessment also highlighted issues that might otherwise have been forgotten, especially in a stressful situation. Parents expressed this with various comments, such as:

*This really ticks all the boxes that need to be discussed. Because, obviously sometimes you can only think of one thing or the first thing that is most pertinent to you, you and your family, and the person you are caring for and other things get forgotten*.

(P23-NC)

- *Subtheme 2.2: Improved communication*

The CSNAT (Paediatric) improved communication between parent and the palliative care service providers or team. Parents described that the assessment opened a discussion between them and the HPs and enabled the parents to identify their needs and to articulate them.

*It was helpful because it did open up discussions with the team and highlighted some of the areas that I have been struggling with.*

(P14-NC)

- *Subtheme 2.3: Raised awareness on issues including the family unit*

Parents acknowledged the impact of their child’s illness and the parental caring role on the family unit, as well as the necessary solutions.

*It highlighted the fact that I probably need to look at him going into respite care maybe once a month. And that is something I always not wanted to do up until now. But it probably highlighted the fact that for my other children it’s a necessity, so they can have some breathing room as well… So, it helped me to recognise that is something that we need to do.*

(P14-NC)

**Theme 3. *Emotional responses to self-reflection*.**

- *Subtheme 3.1: Prompted self-reflection in a positive way*

The systematic assessment prompted the parent to pause and reflect; *“it made me stop and think”* (P25-NC) was a common experience. The questions offered the opportunity for a pause in the daily routine and for them to re-assess the caring situation. Parents commented positively on how this experience affected them.

*I think it was helpful because it just made me think more about [child name]’s needs, my needs and if they are being addressed and how they are being addressed. So, sometimes we just don’t stop to think about all these questions or issues that are raised through this program. And I think it’s a good process. So especially beneficial for me as a caregiver and a parent.*

(P22-NC)

- *Subtheme 3.2: Elicited feelings of confrontation*

For other parents, this triggered a sense of confrontation, and they admitted that *“it’s hard and when you’re reading the questions you have to try and think honestly about how you feel inside”* (P09-C). The parent explained that the avoidance of this confrontation represented a way of coping with the necessary daily life tasks.

*Well, it’s just the whole thing. When you read the questions, it’s not like you are trying to blank out the illness, it’s just, just hard dealing with your feelings and worries, I suppose. I am no different to everybody else, you try to bury your feelings and worries because you still have to go on with other things.*

(P09-C)

- *Subtheme 3.3: Enabled a different perspective*

Reflecting on their needs enabled the parents to take a different perspective, which was expressed as *“just made you think outside the box”* (P12-NC). This process helped them to analyse and understand their thoughts and feelings.

*the questions open up different pathways for your thoughts as well. I think. So, you know it helps you to sort of break down your own thoughts and to, you know, go more into depths of why you are thinking the way you are thinking.*

(P08-C)

- *Subtheme 3.4: Helped themselves and others*

One parent felt that the process of reflection helped in her search for meaning in the serious illness of her child and spoke of positive feelings that emerged despite the sadness.

*At the end of the day I actually realised that even though I am in that situation I actually still feel blessed and that there is actually a lot more that’s positive that’s coming out of this. And it’s not all just bad and sad and so and so. No, I really think it was good for me, personally for me, myself.*

(P02-C)

Likewise, participating in the research was seen as an opportunity to help other parents in a similar situation and comforted the parents that their situation and care experience contributed to something meaningful.

*I see that as a positive in providing information to you. So, that hopefully that feeds back and it provides help to others. And that’s my ultimate goal. I know in the end, that, we might not be able to help [child name]. But, you know, the end goal for me is, if I can, if something good comes out of this it is going to be that we’ve helped inform others about our situation and about what areas are lacking and what can be improved.*

(P08-C)

**Theme 4. *Validation and empowerment*.**

- *Subtheme 4.1: Validation of parents’ needs and role*

Parents appreciated the added comprehensiveness of the assessment and found that it allowed for a different level of acknowledgement and validation of their needs, including their caregiver role:

*This is on a completely different level. And this is about acknowledging and diving deeper and getting me to do some self-reflection. And, and knowing and acknowledging the issues that I’ve got. I think it’s been invaluable; I think it is fantastic and very different.*

(P24-NC)

- *Subtheme 4.2: Established own strategies*

This systematic assessment also encouraged the parents to develop their own strategies, and they adhered to the mutual action plan they had discussed during the assessment.

*But I did start one thing that we discussed on there, which was the meditation. So, I definitely started doing that after the assessment.*

(P21-NC)

*I haven’t seen this psychologist for three months. And I thought I didn’t need to. And then when I went and saw her, I realised how much I had to talk about. So, I can’t leave it that long I have to definitely check in with her every now and again.*

(P21-NC)

- *Subtheme 4.3: Felt reassured to ask for support*

The acknowledgment received during the conversation with the HPs not only made parents’ needs visible, but also legitimised their own needs. Parents’ self-confidence to ask for help increased as they described that they felt reassured to ask, for instance, *“for more respite under the new NDIS [National Disability Insurance Scheme] rollover thing. I have asked for that. So, I put my needs forward with them. And asked for more respite”* (P05-NC).

In addition to taking time for themselves, parents also intended to take care of their own medical needs: *“Just made me realise that some parts like her care I might need to find some support with, just like with time for myself and my medical needs and things like that”* (P31-NC).

- *Subtheme 4.4: Engaged in advocacy*

Some parents mentioned how they engaged in advocacy and that this had been reinforced by the CSNAT (Paediatric) experience. They turned to politicians to raise awareness of their support needs and to increase support for themselves and other families caring for a child with a life-limiting illness.

*I think we just try and look at other avenues to see if we could get things ourselves to speed up the process. I’ve even tried to ring members of parliament and the radio station just to, yeah things like that, just to bring to light the issues what we have. Because sometimes I think if we don’t speak people don’t understand.*

(P28-NC)

**Theme 5. *Received supports responsive to their needs*.**

- *Subtheme 5.1: Parent felt better informed and prepared*

The information and assistance they received from the HPs alleviated parents’ concerns and created a sense of empowerment. They felt better prepared:

*It’s sort of prepared me for what’s going to come and had a plan in place.*

(P07-C)

*having talked to [HP name] and the doctor we know we have plans in action if we need it. So, I feel a little bit more comfort with that.*

(P11-C)

*I guess by having gone through the process and being able to talk to [HP name] about some of my concerns around transition allowed me to have a conversation about what is a clear plan. Who can I turn to in between the process? It helped me to put some steps into place, I guess. Just through having those conversations.* (P15-NC)

- *Subtheme 5.2: Increased confidence and coping*

The improved confidence increased parents’ sense of coping and managing *“I actually do feel stronger about most of the things that I thought I would fail in”* (P02-C) and *“It made me realise that I do a lot and I am taking on a lot and I am managing it quite well”* (P07-C).

- *Subtheme 5.3: Support addressed needs*

The HPs answered parents’ questions, liaised with other services, provided advocacy, and arranged required referrals according to what was identified and discussed in the CSNAT (Paediatric) conversation and action plan. Parents found the provided support to be very helpful:

*She has been able to answer a lot of the questions I had and find the answers that I needed, by going through that form. So, it’s been very helpful. And I am very thankful that you’ve done it.*

(P11-C)

*Obviously, [HP name] and [HP name], anything that I had issues with or that is, you know, I needed help through what we’ve worked out from the questionnaire that they have been able to help, which has been amazing.*

(P23-NC)

The following comment seems to sum it all by reflecting on the uniqueness of this approach, which is truly centred on caregivers:

*Well, I just want to say, thank you very much, really. Because I think that’s something that is, all the year that we had, so many things, we got bombarded with people who want to do surveys and from the health department or, you know, I get calls all the time. But this one seems to be the one right for us and that made it, again, reassuring that somebody is out there trying to understand, or trying to find out where we are coming from.*

(P08-C)

## 5. Discussion 

This study examined the feasibility of using the CSNAT (Paediatric) in the paediatric palliative care setting from the perspectives of parent caregivers of children with life-limiting illnesses. All study participants agreed to be interviewed, and it is apparent that parents appreciated the value of this systematic approach in engaging them in conversations about their needs, priorities, and solutions. They felt it gave them the opportunity to consider and express needs and identify what is important to them in a timely manner where possible. Parents welcomed the focus on their individual situation and hoped that this research would increase the awareness of service providers and policy makers to create a positive change. 

The use of the CSNAT (Paediatric) facilitated the identification of the parents’ support needs, where over 60% reported the following needs: having time for yourself in the day (direct support for the caregiver); practical help in the home (direct support for the caregiver); knowing what to expect in the future when caring for your child (enabling support for patient); financial, legal, or work issues (direct support for the caregiver); knowing who to contact if you are concerned about your child (enabling support for patient); looking after your own health (direct support for caregiver) [12]. It is worth noting that parents needing direct support for themselves featured high on this list, as is supported by the quotes in this article. 

### 5.1. Gaps in Supportive Networks

While clinical services and the support they can offer was affirmed by parents, these parents were left wanting in other areas of practical, psychosocial, and emotional support. This is echoed by a recent independent review of adult palliative care services in WA that used a cross-sectional consumer survey of quality indicators to respond to the six priorities of the WA End of Life for developing and improving palliative care services across WA. Of the six priorities, quality indicators for Priority Four (families and carers are supported) lagged behind the others [19]. Family carers reported not being well supported before and after bereavement by palliative care services. Emotional support and the ability to discuss worries and fears were not adequate, and 40% felt that they did not receive as much support as they wanted from palliative care services during the patient’s illness, and 50% did not after the patient’s death [20]. By way of contrast, responses to Priority Six (the community is aware and able to care) gave high ratings to the care provided by informal networks. Over 90% of respondents relied on the community (family/friends/neighbours/community organisations) to support them before and after bereavement and reported that this informal support was helpful in attending to practical, social, emotional, and spiritual support needs [20].

Another key finding of the independent review was the lower standard of care for non-cancer conditions across all six priorities, dubbed as one of the ‘loser’ groups in palliative care service delivery, thus highlighting the inequity in care [20]. This finding was also echoed in this paediatric study, where there were higher levels of unmet needs for the non-cancer group, as reported in our first article from this study [12].

While the independent review focused on adult palliative care, it is worth replicating the consumer survey on quality indicators for the paediatric population. In fact, the more recent Western Australian Paediatric Strategy for End-of-Life and Palliative Care (2021–2028) has reiterated the need to consolidate the two priorities related to family carers and the community for the paediatric population [21]. Building blocks that will help to achieve Priority Four include the use of standardised assessment tools, ensuring the child’s family has equitable access to support and respite, and services are urged to look at ways to improve opportunities for consumers to support consumers. Priority Six has building blocks around engaging the community to care, including volunteer models and the development of compassionate communities.

Hence, where our study group was left wanting is within the realm of support from their naturally occurring social informal networks or circles of care [22]. If these informal networks or the inner circles of care are not operational or activated, as expressed in parents’ quotes, then compassionate communities models of end-of-life care need to be bolstered (as recommended by the Paediatric Strategy). This can be achieved by training volunteers from the community to seek the practical and social support the family needs to care for the dying from any age group [23]. Volunteers help families tap into the outer circles of care in their community to enhance their social networks in a sustainable way. A local example is the Compassionate Connectors program, a partnership between the South West Compassionate Communities Network and the WA Country Health Services, where the program has been translated into routine practice by the health service: this is a case of a successful partnership between formal and informal networks [23].

Compassionate communities, as part of the public health approach to end-of-life care, offer the possibility of solving the inequity in the difference in the provision of care by enhancing the naturally occurring supportive networks surrounding the patient and family and through palliative care services building stronger partnerships with supportive networks to transform end-of-life care at home [24,25]. Experts in the field have lamented the reality that, *“although palliative care looks beyond the patient to the family, it rarely looks beyond the family to the community or sees the whole person as an individual-in-community”* [26] (p. 133).

### 5.2. Limitations

We acknowledge the lack of diversity in the study sample where parents were primarily Caucasian women from a metropolitan area.

## 6. Conclusions

Assessing needs is a first step in acknowledging areas where parents need support. Formal and informal networks need to work together to provide a platform of psychosocial, emotional, and existential support that is sustainable in people’s own communities. Given that equity in end-of-life care provision is a goal of government policy [27], inequity arising from disease type should be addressed within the health system. Inequity arising from inadequate social support must be addressed by local communities. Both aspects are taken into account by a public health approach to palliative and end-of-life care. Hence, the distinctive focus of this public health approach is that it views the community as an equal partner in the long and complex task of providing quality healthcare at the end of life [20].

## Figures and Tables

**Table 1:** Summary of themes and subthemes.

Theme	Subthemes
Theme 1. Caregiving challenges, perceived gaps, and feelings of isolation	Mental impact on parents and perceived gaps in support Feelings that parents come last Frustration with inadequacies of external providers Care lacking a psychosocial focus and feelings of isolation
Theme 2. Practicality and usefulness of the systematic assessment	Straightforward form and approach—structured and comprehensive
	Improved communication
	Raised awareness on issues including the family unit
Theme 3. Emotional responses to self-reflection	Prompted self-reflection in a positive way
	Elicited feelings of confrontation
	Allowed a different perspective
	Helped with a sense of meaning
Theme 4. Validation and empowerment	Validation of parent caregiver’s needs and role
	Established own strategies
	Felt reassured to ask for support
	Engaged in advocacy
Theme 5. Receiving support responsive to their needs	Felt better informed and prepared
	Increased confidence and coping
	Felt received support addressed needs

## Data Availability

Ethical approval precludes the data being used for another purpose or being provided to researchers who have not signed the appropriate confidentiality agreement. Specifically, the ethical approval specifies that all results are in aggregate form to maintain confidentiality and privacy and precludes individual level data being made publicly available. All aggregate data for this study are freely available and included in the paper. Interested and qualified researchers may send requests for additional data to Samar Aoun at samar.aoun@perron.uwa.edu.au.
